# ‘The Pope’s own hand outstretched’: Holy See diplomacy as a hybrid mode of diplomatic agency

**DOI:** 10.1177/1369148118772247

**Published:** 2018-05-18

**Authors:** Jodok Troy

**Affiliations:** 1 Department of Political Science, University of Innsbruck, Universitätsstraße 15, 6020 Innsbruck, Austria and Visiting Scholar 2016-2018, The Europe Center, FSI Institute for International Studies, Stanford, CA 94305, USA

**Keywords:** agency, diplomacy, English School, Holy See, practice, Pope, religion

## Abstract

The unconventional nature of Holy See diplomats rests in the composite character of their ecclesiastical role as the Pope’s representatives and their legal diplomatic status and commencement to ordinary diplomatic practice. Holy See diplomacy is a form of conduct created by a set of mixed secular and religious standards in which agents are guided by practices. I locate this argument within a classical English School and a conventional understanding of practice, diplomacy, and agency while incorporating understandings of the diplomat as a stranger. The article situates a Holy See diplomat’s mode of agency as a hybrid one by nature, located at the intersections of political and religious modes of agency and substantial and relational conceptions of international politics. I probe this conceptual framework of hybrid agency by analysing episodes involving papal diplomats in turmoil-ridden historical episodes, and correspondence with informed agents.

Pope John XXIII (1966: 106) referred to the role of the nuncio (the papal ambassador) as ‘The Pope’s own hand outstretched’ to the world of states. Popes before him made similar statements, and the ones after him keep echoing variations of it. The acclamation for the expansion of diplomatic missions, for instance, is a common pattern of the pope’s annual addresses to the diplomatic corps accredited at the Holy See (‘HS’). Although the papacy is one of the oldest participants in the international society of states, its diplomatic engagement may perplex, as international society is assumed to be a secular compound. Still, the trajectory of the papacy’s diplomatic entanglement in international society has been constitutive in forming the diplomatic system. Scholars of diplomatic history and diplomatic practitioners have long since acknowledged this relevance of the papacy (Barker, 2006: 32; Der Derian, 1987b; Kerr and Wiseman, 2013: 25, 55; Nicolson, 1977; Plöger, 2005; Rennie, 2013; Roberts and Satow, 2011). Recently, this relevancy has been illustrated yet again by a cable from the American embassy to the HS to Washington after the election of Pope Francis:

despite the disparity in size, governance, and history, we are both global powers, with global interests and influence. From many points of view, the HS is unique to the world in its ability to pursue its own agenda. The Vatican, with its diplomatic relations […] is second only to the United States. (Mastrolilli, 2017)

Diplomats are aware of the scope and influence of the HS diplomatic trajectory, but International Relations tend to set aside the HS diplomatic trajectory and its societal practices as historical footnotes or proxy variables.

By placing the diplomatic entanglement of the HS in international politics to the historical backburner, international studies disdain insights with grave consequences. Today, the Church’s members and officials are increasingly from all over the world and inter-religious dialogue, for example, becomes important as part of the engagement in political and social policy issues under the aegis of diplomacy. Diplomacy itself is a key part of this transformation which offers expanded space for actors engaging in diplomacy (Constantinou et al., 2016). HS diplomacy transfers its religious and political positions well beyond its religious constituency, which is a common feature of the global outreach of religious institutions (Marshall, 2013). Yet unlike other organised religions, the HS global outreach is highly institutionalised, rests on formal diplomatic representations around the globe equal to embassies and acknowledged by international law. This entanglement between the HS and the international sphere of states generates the strange case of agents that are simultaneously clergy and mirror their secular counterparts. Their very existence and practice are an example of how religious and political entanglements in the international realm ‘generate creative, dynamic, and hybrid modes of social and political agency’ (Agensky, 2017: 21; see also, May et al., 2014; Sheikh, 2012; Thomas, 2000; Wilson, 2014).

Whereas ordinary diplomats are serving the goals of a territorial unit, the unconventional nature of HS diplomats is nested in the hybrid character of their ecclesiastical role as the Pope’s representatives and their legal diplomatic status and commencement to ordinary diplomatic practice. Their papal patron’s global perspective renders them unusually dedicated to influencing the conditions beyond their principal’s immediate possessions (such as a territory). This standing places them at an advantage in an international society in which such contextual factors are centrally important. This advantage is reinforced by the degrees to which HS diplomats constitute a combination of transnational identity and national interest. This difference is obvious when looking at instances of how papal diplomats mediate between a conventional approach and the approach of the HS. Between ‘its supernatural mission of salvation and the mundane reality of world politics’, the institution of the papacy ‘remains a singular and surprisingly vital factor in the international scene’ (Conway, 1979: 474). The agents of papal diplomacy are thus a puzzling case of how to merge the aspirations and expectations of a religious principal with a conventional approach of diplomatic practice.

Given the religious nature of its principal, its ordained agents, and international society’s requirements for diplomatic standards and practice, papal diplomacy cannot be like ordinary diplomacy. Papal diplomacy is the result of a religious and political entanglement that generates a hybrid mode of agency of which this article proposes the constituents of its diplomatic practice. This argument is located within a classical English School understanding of practice, diplomacy, and agency while incorporating constructivist understandings of the diplomat as a stranger. The article situates HS diplomat’s mode of agency as hybrid, located at the intersections of political and religious modes of agency and substantial and relational conceptions of international politics, an angle that has not been applied to HS diplomacy by scholars of International Relations and diplomacy.

After a review of the literature on the HS in international society, the next section illustrates the hybrid mode of diplomatic agency. This hybrid diplomatic agency resolves questions at the intersection of substantialist and relational conceptions of international relations and political and religious modes of agency. HS diplomats act on behalf of a religious and a political entity and logic in religious and secular environments. The next step unpacks the historical trajectory of the HS diplomatic practice. The article draws on a hybrid mode of agency by illustrating how the different dimensions and conceptions matter in blueprinting a comprehensive picture of HS diplomacy. The remainder of the paper demonstrates how the hybrid mode of agency varies by degree. The framework of hybrid agency is then probed by analysing incidents involving papal diplomats in turmoil-ridden historical episodes and unstructured interviews and correspondence with informed agents. This section teases out points of broader interest and illustrates the complex entanglement of the religious and political. The conclusion locates the findings in the context of the theoretical framework, the general political interest in the Church, its entanglement in international society, and points out future avenues of research.

## Holy See diplomacy: Hybrid by nature

Notwithstanding recent studies on diplomacy and practice, the claim of diplomacy’s resistance to theorising still resonates in the literature (Jönsson and Hall, 2005; Neumann, 2003). This resistance is even more so when International Relations theory is confronted with the agents of a religious transnational actor. There is a solid body of literature that situates the HS and the Catholic Church in an international context (Abdullah, 1996; Barbato, 2013; Cardinale, 1976; Graham, 1959; Hanson, 1987; Kurth, 1993; Martens, 2006; Murphy, 1974; Rotte, 2007). This literature focuses variously on soft power (Byrnes, 2017; Sommeregger, 2011; Troy, 2010), international organisations (Abdullah, 1996; Araujo and Lucal, 2004a, 2004b, 2010; Chong and Troy, 2011; Leustean, 2013; Neale, 1998), bilateral relations,^1^ international law (Casaroli, 1981; Morss, 2016), the HS and the church as transnational actors (Barbato, 2013; Ryall, 2001; Vallier, 1971) and their mobilising power (Barbato, 2016; Turina, 2015), the Pope as chief diplomat and moral authority (Hall, 1997), or theological explanations of political outcomes where the Church has been involved in peacebuilding efforts (Cortright, 2008: 200–203; Riccards, 1998).

Most of this literature describes the HS diplomatic structure and community. But the studies that provide insights into the practices of the HS diplomatic service are case studies in which members of the diplomatic service or special envoys achieved a conflict settlement (Laudy, 2000; Princen, 1987, 1992; Schelkens, 2011). Questions about power, statecraft, and how to put the HS diplomatic apparatus in the context of International Relations theory are largely absent in the literature, nor are there any comprehensive conceptual outlines of its diplomatic agential practices. The few exceptions that do so focus on the actorness of the HS as a principal actor, rather than on its agents (Barbato, 2013; McLarren and Stahl, 2015; Neumann, 2011).

Given the formalised and institutional setting of HS diplomacy, this section sets out a conventional conceptualization of its diplomatic apparatus and practice. Like other actors that practice ordinary diplomacy, the HS diplomatic practice follows international formalised and recognised rules. The rather conservative education and public restraint of its members resemble what is usually termed as ‘high politics’, in the sense that it is a realm of senior statesmen. For the sake of foreign policy assets, agents were supposed to practice diplomacy as an art. Certainly, the ideal type of diplomacy Realists had in mind was already in their own time more of a nostalgic sentiment than a realistic option (Bessner and Guilhot, 2015). Yet in the case of the HS diplomatic service, this understanding of diplomacy as an asset still applies, as its diplomats face less constraint by democratic processes and public opinion than their secular peers (Morgenthau, 1978: 525–531). In sum, a conventional conceptualization is warranted by the fact that the HS perceives its diplomatic service in realistic terms as an asset for its foreign policy (Cahill, 2017).

However, given the puzzle outlined in the introduction, a conventional notion of HS diplomacy needs a contextualization of diplomacy as the practice of religious agency if it is to be understood as a hybrid mode of agency. This context requires moving beyond ‘explaining’ practices by ‘naming’ them (Sending et al., 2015: 9) or describing rules and institutions. Rather, a hybrid mode of agency needs to differentiate why agents do what they do, based on their self-understanding as ordinary and ordained diplomats and how the two modes merge. Focusing on the institutional setting and agency of the HS diplomatic service as a practice involves an approach ‘in which action reflects the ideas, cultural contexts, identities, and shared understandings of individual and state actors’ (Green, 2014: 1). Such an approach focuses on agency, ideal types, self-justifications and discourses by interrogating practices in order to discern their normative content (Navari, 2009: 3).^2^ Rather than a form of behaviour or an individual habit, here diplomacy is understood as a form of conduct created by a set of societal standards in which agents are guided by practices (Navari, 2011: 626–627). Practices are ‘bundles of rituals, words and even physical placements, to which autonomous individuals look as guides for appropriate social behaviour’ (Navari, 2011: 613). HS diplomacy, then, is a form of conduct created by a set of mixed secular and religious standards in which its agents are guided by practices, rather than caused by them.

**Figure 1.**
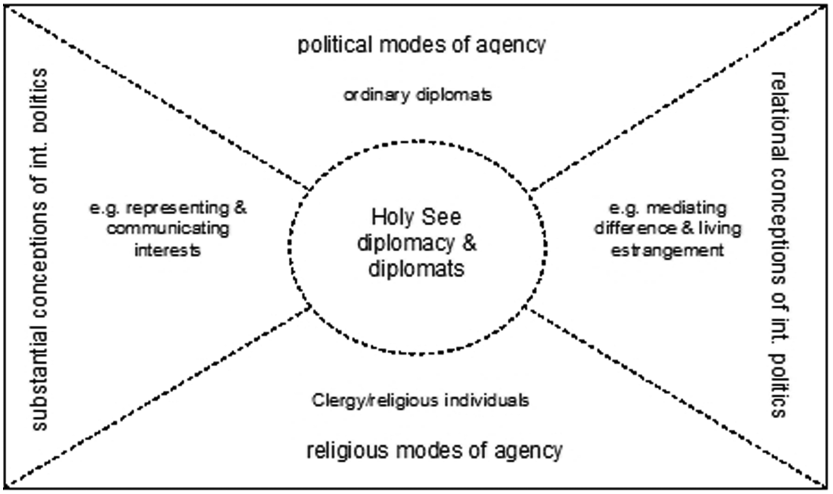
Hybrid mode of Holy See diplomacy

Studying the ‘diplomatic community *itself*’ (Wight, 1966: 22) and the practice of the ‘pope’s men’, rather than ‘the’ pope or ‘the’ HS, reveals supplementary insights to studies focusing on the actorness of the HS or studying ‘the’ diplomacy of this actor. The advantage of this approach is researching diplomacy not only as a category of analysis, but also as one of explanation of what ‘the pope’s men’ are doing. The title ‘nuncio’, for instance, is one who announces the will of the pope but in practice he does much more. In broader terms, conceptualising HS diplomacy as a hybrid mode of agency comprises four dimensions based on the religious and political modes of agency and substantialist and relational conceptualizations of international politics (see Figure 1). HS diplomacy cumulates at the intersection of all four dimensions. It is neither only about ordinary diplomats representing and communicating the HS interests or mediating difference between political principals, nor are its agents simply members of clergy and religious individuals who participate in faith-based diplomacy (Johnston, 2003; Troy, 2008).

This conceptualization of HS diplomacy as a hybrid mode of agency builds on Agensky’s (2017: 21) instruction that religious and political entanglements ‘generate creative, dynamic, and hybrid modes of social and political agency’. Ignoring ‘the relational dimensions of religion and international politics encourages neglect of key moments in the production of religious and political identities and practices, as well as the implicit normative position taken by doing so’ (Agensky, 2017: 3–4; see also Wilson, 2010). International Relations theories often rest on substantialist conceptions of politics that build on autonomous acting subjects. As such, they frame diplomacy as an instrument of foreign policy and focus on representing and communicating the principal’s interests.^3^ Hence, they set aside what logic the subjects follow (Jönsson and Hall, 2005: 14),^4^ let alone on what identities and practices those logics rest. A turn to the English School notion of practice does not suggest a complete shift of the analytical focus to a relational conception. Doing so rather adds a complementary approach since any institutionalisation at the ‘cognitive level entails the development of a common language and intersubjective structures of meaning and interpretation of words, actions and symbols’ (Jönsson and Hall, 2005: 40).

In other words, an analysis of diplomacy needs to turn to the question of how ‘what one knows in diplomacy and what one makes of that knowledge depends on what one understands diplomacy to be – and vice versa’ (Constantinou, 2013: 142; originally in emphasis). This question is important in addressing diplomacy, particularly its agents’ problems of living estrangement between them and the host society if international politics is to be understood from a relational angle. The task of diplomats is to act in and re-act to the macro and micro surroundings, other than the well-known ones of their own (national) community. Diplomacy is thus also about ‘how we can live together in difference’ (Constantinou, 2013: 142; originally in emphasis). Diplomats epitomise the human desire to live separately and to maintain relations with others. Being a stranger and living estrangement are thus inherent parts of being a diplomat (Der Derian, 1987a, 1987b; Sending, 2011; Sofer, 1997). This status fulfils social and political functions such as providing the necessary distance for negotiations in a substantialist conception, which takes diplomacy merely as a functional asset of foreign policy.

This substantialist conception is a permeating condition for HS diplomats, yet in their case diplomacy becomes a hazardous endeavour. They do not only represent the HS but also the Catholic part of the host state’s society qua their institutionalised religious affiliation (Cardinale, 1976). Hence they cannot, by definition, remain strangers in their host states. Whereas HS diplomats are part (at least the Catholic part) of the host society, ‘secular’ diplomats largely remain strangers.^5^ What is more, HS diplomats are ‘foreign nationals’ representing the interests of a political entity which is not their own from the perspective of inter-state relations. Conventional substantial approaches of diplomacy defect this intersubjective impetus of how people can live together in difference. Instead, they focus on diplomacy as a dependent variable, referring to the macro social structure (see for example Kissinger, 1995, 2014) or the micro structure in formal terms (Gould-Davies, 2013; Neumann, 2002; Pouliot and Cornut, 2015). However, diplomacy is always an intersubjective enterprise in which ‘[d]ifferent people with different social traits, will report different things’ (Neumann, 2010). Not only do they report different things, but they do things differently.

## Contextualising the Holy See diplomatic trajectory

There are at least three challenges that any investigation of the HS diplomatic service and its practices faces. First, there is a lack of data on diplomatic and political micro practices.^6^ As large as the diplomatic apparatus of the HS is, it is the Pope on whose person the public and academic focus rests.^7^ This section tackles this problem by turning to unstructured interviews and correspondence with nuncios, informed agents, and an examination of historical episodes involving three prominent nuncios in the 20th and 21st century. Second, the HS dedicates a great deal of resources to internal matters of faith. An analysis of diplomatic practice thus cannot put aside theological explanations in the course of analysing different self-understandings. Third, diplomatic achievements and failures are hard to evaluate, as they are conducted behind closed doors and tend to remain secretive.^8^

It is thus inevitable that any study, other than a single case study, remains in large portions abstract and eventually must point out the necessity of further field studies. In its first part, this section builds on the legal and historical trajectory of the HS diplomatic practice. Based on such a conventional account of HS diplomatic practice, the second part weaves together the education of HS diplomats and conceptualizations of their practice in compliance with the tasks of ordinary diplomats. From a perspective of substantive conceptions of agent’s missions and under the lens of a political mode of agency, HS diplomats appear like other diplomats. However, the remainder of the section illustrates that how diplomatic tasks are accomplished depends on how diplomats act on the ground based on intersubjective practices and standards of excellence within their communities. Those practices and standards of excellence render them not only under a political but also religious mode of agency.

HS diplomats are engaged in traditional secular diplomacy such as the aspiration of good relations between the HS and the host nation. In doing so, diplomats represent the pope as the head of the Catholic Church and not as the head of the Vatican.^9^ The legal embodiment of the Church is the HS (the papacy) which is an international personality in international law. In terms of international law, the ‘State of the Vatican City’ and the HS are different entities. They are linked by the personal-union with the pope, the supreme head of the Catholic Church and the Vatican City. The foreign relations of Vatican City are managed by the HS, which is subject to international law. In operational terms, the management comprises the HS Secretary of State and its subordinate, the Secretary of Relations with States.^10^ In 2017, Pope Francis created a third section, the Secretary of States (‘Section for the Holy See diplomatic staff’), which is exclusively dedicated to the work of the nuncios (Agasso, 2017). Its installation is yet another indicator of the continuing importance the Church attributes to its diplomatic engagement.

Ever since the development of the modern international system, its entities needed a way to communicate in a structural establishment marked by the gap in the relationship between and within political entities (Morgenthau, 1956: 30; Sharp, 2009: 293). It was then that diplomacy became a formalised interaction between political entities. From its beginning, the HS participated in this formalised interaction. This was not only because (after the Lateran Treaties in 1929) the HS acquired a formal territory, but because it pressed to preserve its normative influence in the international society.^11^ First attempts of regulated and institutionalised diplomatic activities of the HS date back to the 15th century when it became necessary to regulate rights and obligations between the Church and various political entities. The institutionalisation of HS diplomatic practice was not the result of one particular pope or period of history but evolved impulsively. In modern times this impulsive character of the institutionalisation of HS diplomatic practice became visible as the diplomatic apparatus drastically expanded during the regency of Pope John Paul II (1978–2005).^12^ Today, the HS has diplomatic relations with almost all countries around the globe and newly consolidated nations keep seeking recognition from the HS (Sommeregger, 2011: 132–135; The Economist, 2007).^13^

The conduct of the diplomatic apparatus such as the maintenance of nunciatures is regulated in the Code of Canon law.^14^ Nunciatures regularly report on matters of the Catholic Church in host countries, and they seek unity between the HS and the local Church. Since the Congress of Vienna the Doyen (the senior ambassador) of the diplomatic Corps has been recurrently the nuncio, at least in Roman Catholic countries.^15^ In terms of international law, nunciatures hold the same status as embassies. Canon law offers a range of functions for papal legates, most of them dealing with internal matters of the Church such as assisting local bishops. These internal matters include traditional clerical duties, such as the appointments of bishops, which influence domestic and international politics as the contested cases in the People’s Republic of China illustrate (Wee, 2014). However, that clerical task routinely propels critics to point out that canon law suggests that the nuncio is a papal watchdog to supervise the local Catholic Church (that is, the dioceses).^16^

The HS diplomatic envoys and nunciatures had their high-days in the medieval ages and early modern era. Later, the popes themselves took over the diplomatic agenda (Kent and Pollard, 1994b), applying traditional, symbolic, legal, and representational instruments of diplomacy in their every-day conduct. Scholars of diplomacy and International Relations thus partly attribute the evolution of the diplomatic culture as we know it today to Christendom, noting a Christian influence on the diplomatic culture up to the present day (Meyer et al., 1997: 174; Neumann, 2012: 310).

### Conventional education, conventional practice?

The *Pontificia Accademia Ecclesiastica*, founded by Pope Clemens XI in 1701 in Rome, cultivates the religious influence on ordinary diplomacy. In modern times, it played an important part in professionalising the diplomatic service of the HS, as it consolidated its bureaucracy, particularly in the first half of the 20th century (Alvarez, 1989). The academy proved to be the prime educational site for future high ranking Church officials and popes. Popes themselves influenced the Church’s domestic and international reputation within the academy. Pope John XXIII (1958–1963), himself not an alumnus of the academy but a respected diplomat, was a powerful influence, as were his successors Paul VI (1963–1978) and John Paul II (1978–2005). The latter, due to his interest in foreign policy, was a frequent guest at the academy. The task of the academy is to acquire and educate young clerics from the ‘Roman-cultural’ environment for future tasks in the secretary of state or in nunciatures.^17^ Since the foundation of the academy its presidents were largely Italians and its students largely from outside Europe (Kracht, 2011: 987–989).^18^

Not least due to its geographical location, for the most part the academy remains Italian in character and with the exception of a few outsiders, the Secretaries of States, its most prominent alumni, have been Italian (Kent and Pollard, 1994a: 12).^19^ At his first address to the community of the academy, Pope Francis called for the education of future papal representatives as priestly ministers, a task that demands ‘great inner freedom’ and the abandonment of ‘careerism’.^20^ Given its 300 years history and influence on the education of Church officials, the question of whether the *Pontificia Accademia Ecclesiastica* aptly is an academy or a college remains an open one (Kracht, 2011: 989–990; Oliveri, 1982).^21^

The existence of HS diplomats over the course of centuries and their conventional training illustrates that the papacy is not unique compared to other diplomatic actors in the international realm. It is yet another example of institutional accommodation and structural modernization of the diplomatic service, with the aim of establishing a traditional and functional diplomatic apparatus (Alvarez, 1989: 248). In this regard, HS diplomacy is a performance of a host of practices that follow the general practices of a diplomatic community (Sharp and Wiseman, 2007) such as maintaining permanent representatives in states and international organisations. Those practices are patterned as they entail particular rules (for instance, the ones laid out in the Vienna Convention on Diplomatic Relations), they are competent because diplomats are educated practitioners and the education is institutionalised, and they rely on institutionalised background knowledge. Accordingly, as diplomats, the agents of the HS tend to be skillful in their practice.

The practice of HS diplomats also weaves together discursive and material worlds. Its diplomats engage in a dialogue that weaves together claims of the Church’s moral authority with material worlds such as the vast bureaucratic infrastructure of the HS and its nunciatures worldwide.^22^ They are essentially legally recognised agents in an international society that grounds its dialogue in diplomacy (Watson, 1991). They are trained as diplomats, they are experts in the tasks and missions assigned to them and based on their religious affiliation they are nominally also moral experts. This phenomenon becomes obvious in looking at how diplomats pursue and engage in different foreign policy goals. Diplomats participate in shaping and acquiring possession and milieu goals of foreign policy (Ryall, 1998; Wolfers, 1962: 67–80). Reflecting the actor’s national interest, possession goals aim at the enhancement or preservation of something the actor values. Milieu goals, on the other side, aim to shape the conditions beyond the actor’s immediate possessions. Seen from the perspective of the HS, priority is assigned to milieu goals on the grounds of its universal salvic mission. A marker of the emphasis on milieu goals is the HS gaining of a seat as a permanent observer at the United Nations.

In line with his ‘bolder’ vision of diplomacy (Povoledo, 2013; Yardley, 2014), Pope Francis called for a more intense dialogue with Islam and a renewed diplomatic discourse with states that have no official ties with the HS. The HS has no official diplomatic ties with China or North Korea but in both cases, there are various movements on the side of the HS, either to improve relations or to establish institutionalised relations.^23^ The engagement in milieu goals illustrates the weight the Church places on the practice and institution of diplomacy. To be sure, pursuing milieu goals is not always successful or appreciated by other actors in the international society. The HS, for example, was blamed for triggering the wars in the Balkans in the 1990s by prematurely recognising the independence of Catholic states (Allen, 2014: 7, 152; Byrnes, 2001: Chapter 4; Grant, 1999: 178–179).

The participation of the HS in international organisations is not least due to increasing pluralism in international politics, which calls for an institutionalisation of diplomacy (Mayall, 2007: 2). Since the pope has no ‘divisions’, as Josef Stalin once allegedly remarked, establishing conventional formal relations with governments is one way to maintain geopolitics based on the sheer numbers of humans involved (Agnew, 2010). In this regard, the HS is a rare example^24^ of a non-state actor with diplomatic privileges, recognised by the majority of states in the international realm, of which diplomacy is a foundational and institutionalised pillar (Bull, 2002; Bull and Watson, 1984; Jönsson and Hall, 2005; Wight, 1997). It is therefore no surprise that the HS supported the development of modern international governmental organisations (Chong and Troy, 2011; Leustean, 2013; Melnyk, 2009; Tomasi, 2017; Wuthe, 2002).^25^

As principal, the HS seeks to justify its engagement in shaping possession and milieu goals on theological grounds, communicated by its agents which establish the hybrid diplomatic agency. However, although there are policy guidelines for both goals, their implementation process is far from being coherently implemented by the agents, which illustrates the limits of a conventional notion of practice.

## The Pope’s eye, heart, and hand: Ordinary and ordained

HS Secretary of State Pietro Parolin characterises HS diplomacy as ‘human diplomacy’ because ‘real people must be at the centre of all diplomatic action’.^26^ According to Cardinal Sodano (2000: 91) (HS Secretary of State 1990–2006), the goal of papal representation is ‘to bring the leaven of the Gospel to all the complex reality of international relations and to international debates’. The aims of HS diplomats are to serve and represent the interests of the Church, to maintain or establish peace between nations, and the empowerment of international and supranational structures (Sharp, 2009: 133–134). ‘Political support or material aid they will certainly not expect’, reads the statement by the HS mission to the United Nations on the purpose of its agents. ‘What they do seek’, instead, ‘is what the HS, by its very nature and tradition, can offer: orientation and spiritual inspiration that should animate the life of nations and their mutual relationships’.^27^ This spiritual duty is also reflected in the Church’s Canon law:

diplomatic relations of the pope are always carried on in light of [the] spiritual mission, hence legates represent the Holy See […]; civil governments enter into relations not with the State of Vatican City but with the Holy See itself [and] the title given representatives of the Holy See (‘nuncios’ rather than ‘ambassadors’) is intended to underscore the particular nature of their mission. (Coriden et al., 1985: 302)

The ‘Apostolic Nuncio is one of the most characteristic signs of the Pope’s presence in a nation’, as Pope John XXIII, former nuncio to France, summarised it. Moreover, pontifical ‘and national diplomacy have a common basis in frankness and in the constant effort to improve relations. But they differ in their essential characteristics’ (Pope John XXIII, 1966: 105). The nuncio is the pope’s eye, heart and hand in the host state. What Pope John XXIII stated for his position while nuncio in France can be read as the tasks of an ideal-type nuncio:

An eye ever open to serene observation of the moral, religious and social state of France, in order to render a true and objective account to the Supreme Pastor. A heart watchful and sensitive to the needs of the people of Christ, as hungry for spiritual foods as they are for bodily sustenance. The Pope’s own heart indeed, for his mission is not to busy himself with material conquests and earthly interests, and not to follow in the ways of intrigue and hatred, but to observe the law of brotherhood and love. Finally, a hand to point to the right road, a hand which, in the Lord’s name, succours, encourages and blesses. This is still and will always be the Pope’s own hand outstretched, reproduced in the person and office of his Nuncio, in France and in every other nation. (Pope John XXIII, 1966: 106)

This instruction is similar to conceptualizations of ordinary diplomatic modes of agency and conventional notions of practice, which is obvious in both the first and the last sentence of the quote. It stresses the importance of the diplomat’s task of gathering information and communicating interests. It combines the view of diplomats as representing a principal in behaviour (to act for others) and in status (to stand for others) (Jönsson and Hall, 2005: 100). It illustrates the thin line between representation and governing (Mitzen, 2015) when it asserts that the nuncio should point ‘to the right road’. Finally, the instruction indicates that nuncios, like ordinary diplomats, also act on behalf of certain ideas and not only on behalf of governments and principals. Peace has been seen as the most elusive of human ideas, and is indeed an objective in this regard (Jönsson and Hall, 2005: 116; Macomber, 1975: 25), no less so in HS diplomatic practice (Parolin, 2017: xv–xvi). The objective of peace predates the objective of serving the principal (Sharp, 1997: 616) as an asset that mediates between different entities.

This instruction is also an expression of the religious mode of diplomatic agency and as such it is illustrative of the HS hybrid character with regard to diplomacy. Pope Francis, addressing the diplomatic academy, reinforced the main elements of the religious mode of agency in this instruction. In particular, he stressed the need for HS diplomats to keep their spirituality amid their worldly tasks and pointed out that their future life will be inserted in different societal contexts. He also pointed out that HS diplomats need to be examples in their lives, characterised by closure, dialogue, listening but also tangible verbal action. What is more, he stressed that HS diplomats need to serve the evangelization without proselytising as the Church grows by attraction (Pope Francis, 2013; Radio Vatikan, 2017).

### Hybrid modes of agency in practice

The remainder of this section turns to three cases of nuncios in turmoil ridden historical episodes. The cases illustrate that although there are outlines of an ideal-type nuncio such as illustrated above, their practical mode of agency remains a hybrid one as it comes into existence through the interaction of different origins. Although any hybrid mode has its own outstanding characteristics and remains hybrid in nature, it varies in degree. This variance is no different in the case of HS diplomats. The three episodes of the nuncios Joseph Hurley, Jose Laboa, and Fernando Filoni illustrate that the mix of principal steered possession and milieu goals, diplomatic practice, and the self-understanding of the agents add up to a contextualization of the HS diplomatic service and the practices of its members as a hybrid mode of agency.

Joseph P. Hurley from the United States was perhaps one of the most influential yet largely unknown papal diplomats in the 20th century. Hurley originally served in the Japan mission in the 1930s and was a warning voice of National Socialism and Fascism. He served again as nuncio in the 1940s in Yugoslavia and became known for his fierce stand against Communism. Hurley is an outstanding example of a papal diplomat as he resembles many of the aforementioned ideal type features of ordinary diplomats. He was sympathetic towards an approach of diplomacy understood as ‘high politics’ such as secretively operating behind the scenes and keeping a low public profile. Yet he also believed his role to be the ‘Pope’s own hand outstretched’. Throughout his life, he was torn between serving his two masters: the United States and the Church, and rival interpretations over the mission of them (Gallagher, 2008: 1–3). In the early Cold War years, he sided with the US State Department, with which he became disappointed in the 1950s as he found no more ‘convergence between Catholic ideas and U.S. political realities’ (Gallagher, 2008: 196).

The episode of Hurley illustrates how difficult it is to grasp the practice of HS diplomats along the religious model of diplomatic agency. During several stages of his career Hurley, in many respects, might have come close to a conventional diplomat but he remained so in ordinary diplomatic terms. Although he assumed his role as serving church and country, he sometimes placed the mission of the latter over the former. Arguably, in this regard Hurley was a classic example of a power politic driven agent, mediating between different political entities. As Hurley tried to serve two principals, he encountered difficulties in doing justice to both as well as becoming even more disappointed when the two principals parted ways in their goals in the 1950s. The story of Hurley illustrates that HS diplomacy is not always conducted along standardised diplomatic practices. In Hurley’s case, this divergence meant that he drifted from representing to governing, mediated by personal beliefs regarding the political and the religious realm. Yet still, he complied with the art of diplomacy as a form of high politics and certainly was seen as an outstanding diplomat.

Another episode illustrates how a papal agent got caught between the principal’s foreign policy goals and ended up improvising. It is the episode of how Panamanian leader Manuel Noriega was eventually handed over into the hands of US authorities. In 1989, rather unexpectedly, Jose Sebastian Laboa from Spain and nuncio to Panama was faced with the difficulties that serving possession goals bring with them when Noriega took refuge in the nunciature. Without being able to consult the HS at first, the nuncio extended the diplomatic immunity to another house to separate Noriega from his aids. Eventually, Laboa, who had been encouraging anti-Noriega forces before, was able to sway Noriega out of the embassy and into the hands of US forces. He did so through a carefully orchestrated psychological campaign, warning Noriega of the danger of being lynched or by talking about loyalty at the Catholic Mass. All of this practice aimed to convince Noriega that his only realist option was to give up (Kempe, 1990: 398–417; Parmelee, 1989; Rooney, 2013: 169; Rosenthal, 1990; Rother, 1990). In his approach of doing so, Laboa illustrated the behaviour of a classical rationalist driven agent of international society, subtly complying with international law to achieve his goals. However, because Laboa turned to the religious practice of the Catholic Mass attended by Noriega, as a means to practice diplomacy, Laboa also relied on elements only an ordained diplomat can turn to such as the Catholic liturgy.

The episodes of Hurley and Laboa illustrate that, on the surface, HS diplomats operate like ordinary diplomats, face the same challenges, and rely on the same instruments. However, they also know how to use their authority as ordained diplomats. Without an in-depth study into the agents motives, the episodes only hint towards a genuine hybrid mode of agency. We can find this mode in operation in the more recent case of nuncio Fernando Filoni, originally from Italy.

Recalling the mediation of difference and estrangement, Filoni’s case illustrates how HS diplomats are an inherent part of the host society, and not only the Catholic part of it. Filoni was the representative of the Pope in Iraq in 2014 but first became famous for remaining on his post as nuncio in Iraq during the 2003 US bombing of Bagdad (Allen, 2014; Filoni, 2009). Explaining his remaining on in the post, he argued that it ‘was nothing exceptional. To live in Baghdad during the war was a decision in line with the mission of a pontifical representative who … by residing in the countries participates, or rather inserts himself into their life. Our very situation in itself led us to share the destiny of the Iraqi people with all their sufferings, injustices and hopes’.^28^ Filoni’s actions constituted a modality of practice and discourse of a Church of the margins as it is today attributed to Pope Francis’ conception of the Church (Ferrara, 2015; Ivereigh, 2015) and its diplomatic service more broadly. In this regard, Filoni appears as an advocate of a cosmopolitan idea of world society. While staying in the apostolic nunciature in 2003 and justifying his stay, he weaved together claims of religious agency and political agency based on material worlds such as diplomatic representation. The episode of Filoni illustrates what an ideal-type of a hybrid agent of papal diplomacy could look like. It emphasises the need for a diplomat to insert himself into the society of the host nation and eventually to rely on possession goals (such as immunity of diplomatic premises) and milieu goals (for instance, emphasising the universal mission of the Church).

Seen in chronological order of the historical episodes described above, HS diplomatic practice seems to move from a realist system, over the rationalist society, towards the revolutionist world society approach (Wight, 1991). The episode of Hurley illustrates the persistence of the realist tradition of international society as international system. Caught in great power rivalries, Hurley aligned himself with one ideological side, using diplomatic expertise and legal status to influence foreign policy. In doing so, he practised diplomacy as ‘high politics’, argued for on moral grounds. The episode of Laboa and his subtle compliance with international law illustrates renewed rationalist aspirations of international society at the end of the Cold War. Finally, the episode of Filoni shows a modality of a Church of the margins, resembling the revolutionist approach of international society as world society that seeks to set itself against the state system and its secular conventions. The episodes also illustrate that HS diplomats are representatives of an entity that is not their own in terms of inter-state relations. They are born in one country and represent another political and religious entity to a third country. The hybrid nature of their agency is thus a rather convenient fact that waives the inherent strangeness that accompanies ordinary diplomats on their tasks.

## Conclusion

Despite the weakening influence of the Eurocentric Catholic Church, HS diplomacy continues to play a vital role for the Church itself and in international society. In conflicts around the world either a Catholic majority or a significant Catholic minority is at home. Moreover, in a global Church its officials are from all over the world rather than only from the Roman ecosystem. Globally, religion wields an arguably growing influence in international politics (Thomas, 2010), and there is more space for creative diplomatic engagement (Constantinou et al., 2016), both of which are significant trends for the future of understanding diplomacy and international politics. The HS, for example, has found global reach and mobility accompanying globalisation as a vehicle to overcome the traditional territorial and nation bound state (Turina, 2015: 201) without giving away its secular features of diplomatic practice (Parolin, 2017).

A renewed interest in diplomacy is not least propelled by the current Pope and his intermingling in conflicts around the world (Gaetan, 2015, 2017). He refuses to align himself in global politics and continues to expand his diplomatic corps (Franco, 2013; Jones and Mackenzie, 2015). This choice comes as no surprise as diplomacy is constitutive for the participants in international society and thus also the Church: it is a fundamental and durable practice; it is constitutive of the HS in recognising the world of states and the formalised way of maintaining official ties therein; and its patterned structure and agency legitimate its activities in relations to others as it sticks to the formalised rules of conducting this practice. Particularly, the last point illustrates that HS diplomacy is also a form of conduct created by a set of standards in which agents are guided by practices.

HS diplomats, as the three episodes of nuncios illustrate, exercise authority under legal, moral, and expert modalities, weaving together secular and religious discourses and practices. They are essentially and functionally recognised agents in the international society that grounds its dialogue on diplomacy. All three trained as diplomats and experts in their tasks and missions. Based on their religious affiliation, they are nominally also moral experts. The three episodes illustrate how modalities and discourses interact and sometimes trump each other. In doing so, they also illustrate that international society is not a one-way street to a justly governed world society (Wight, 1991: 266).

Early English School scholars put forward the claim that religious institutions are constitutive for international society. Those claims, however, have been picked up only reluctantly in subsequent studies. The entanglement between religious institutions and international society, after all, remains an understudied topic.^29^ The theoretical conceptualizations of power, diplomacy, and international society demonstrate insights into the HS diplomatic conduct while not only framing diplomacy as a category of analysis but also as one of explanation. Diplomacy is a practice because political units need to interact and want to stay apart. In the conduct of international society, this gap is filled by the practice of diplomats, no less so by the ones of the HS. They are even more likely to bridge this gap because estrangement on the cultural and religious level tends to be low but remains intact when it comes to secular duties where social distance remains a condition for pursuing foreign policy goals. The findings of this article suggest a renewed research interest looking into the interplay between the hybrid agency of diplomats and structural issues of international society.

## Notes

1. Especially with the United States (Essig and Moore, 2009; Franco and Flamini, 2008; Rooney, 2013).

2. In broad terms ‘practice’ and ‘institution’ are synonymous (Little, 2011: 176).

3. This framing adds to the marginalisation of diplomatic theory (Jönsson and Hall, 2005: 12–19).

4. Conceptualizations of religion and religious actors suffer the same fate (Fitzgerald, 2011; Sheikh, 2012).

5. Sharp (2009: 84) points out that: “there can be no diplomacy when people are completely separate and, hence, unaware of one another. … It is when people want those relations with one another, but also want to keep apart, that the conditions of separateness are created. And these conditions provide the space in which diplomacy and diplomats work.” On the other side, there are never two states that enter diplomatic relations but a state and a de-facto religious community (since the HS is a legal personality of its own).

6. Depending on the subject, there is a several decades long archival blockade. Although there is a rich body of literature on the correspondence of nuncios, those documents are mainly historically interesting (see for example Koller, 1998). To complicate matters, there is no tradition of memoir literature from nuncios, except of Angelo Giuseppe Roncalli, former nuncio to France (and later Pope John XXIII, 1966).

7. John Paul II’s behaviour illustrated this vividly. His predecessor, Roncalli, was a prominent diplomat before he became Pope John XXIII.

8. One notable exception is WikiLeaks cover-up of diplomatic communiqués.

9. See Canon Law canon 363 § 2 and canon 365. Vice versa, diplomats of other states are accredited at the HS and not at the Vatican.

10. Diplomats accredited to the HS are not physically resident at the Vatican City State (Cismas, 2014: Chapter 4; Köck, 1975).

11. This effort seems to suggest that the HS became a ‘secular’ actor only after the Lateran Treaties, which is not the case. Before those treaties the Papal States did not qualify as the Church in an earlier state-like form. The diplomatic ties then have been only with a selective number of countries. Still, the Church was a hybrid actor even before the Lateran Treaties but not that complex institutionalised in the sense of an institution of international society. See critically Morss (2016).

12. The dates refer to the popes tenures.

13. http://goo.gl/mCzczB.l.

14. Canons 362 to 367; http://goo.gl/K9wuE8.

15. This fact suggests that the nuncio could not have been the doyen at the Congress of Vienna if the Papal state and HS were not part of the international society. In many (mostly Catholic) European countries this is still the case.

16. See, for example, canon 363, § 1 and canon 364. Revising the Canon law, John Paul II put more emphasis on ecclesiastical matters to conserve Christian unity (Feldkamp, 2010: 131; Morss, 2016; Turina, 2015).

17. Clerics with an accomplished academic study (at least a theological doctorate) and practical ministerial experience are trained at the academy in administrative and canon law, diplomacy, history, and at least two more foreign languages in addition to their mother tongue and Italian. Confidential correspondence with HS diplomats (emails to the author).

18. http://www.vaticandiplomacy.org; Alvarez (1989: 238)

19. Rome, seen this way, continues to be a centre of world politics when it comes to religious engagement in foreign policy (Petito and Thomas, 2015).

20. http://goo.gl/cjBcSG. Radio Vatikan (2017).

21. This distinction becomes obvious at its curricula. Students are recruited from the ‘secular clergy’ (that is, deacons and priests) and stay at least two and at most four years at the academy. Their education covers several aspects: (1) *Courses* in the academy are taught by the personnel of the secretary of state on (a) papal diplomacy by the undersecretary of the section for relations with states, (b) diplomatic style, (c) international law, and (d) history of papal diplomacy. (2) A one month *internship* at the secretary of state during the summer. (3) *Meetings* with the leaders of ministries at the academy. (4) *Language* education during the year and during the summer months. The education takes place at papal universities with the final achievement of a doctorate within a subject of choice. A degree in canon law is required. If this result is not achieved, an adequate canonical education is required. Of no lesser significance for the education is the fact to be permanently based at the academy, under the leadership of someone who usually has been a papal nuncio or a member of the diplomatic service of the HS. Prominent examples of the reading list are: Pontificia Accademia Ecclesiastica (2003) and Prudhomme (2004). Confidential correspondence with HS diplomats (emails to the author).

22. For this conceptualization of practice, see Adler and Pouliot (2011: 6–7) which is different from the one of the English School that stresses the telic notion of practice. A conventional, causal approach of practice is helpful to capture the conventional strings of HS diplomacy.

23. There are, for example, various attempts of the HS to establish formal diplomatic ties with China. Confidential conversation with a former religious non-governmental organisation official.

24. The others are the Sovereign Military Order of Malta and the EU (Bátora and Hynek, 2014).

25. This position puts the HS in a dubious light when cooperating with dictatorships on issues such as birth control (Neale, 1998).

26. http://goo.gl/cybvi2.

27. https://holyseemission.org/contents/mission/our-history.php; Radio Vatikan (2017).

28. http://goo.gl/0vB7DY; Allen (2013).

29. Martin Wight (1979), for example, did so. See also Diez (2017).

## References

[bibr1-1369148118772247] Abdullah Y (1996) The Holy See at United Nations conferences: State or church? Columbia Law Review 96(7): 1835–1875.

[bibr2-1369148118772247] Adler E Pouliot V (2011) International practices. International Theory 3(1): 1–36.

[bibr3-1369148118772247] Agasso DJR (2017) The Pope creates the third section of the secretariat of state: It deals with nuncios. La Stampa, 20 11 Available at: https://goo.gl/8UN4mf (accessed 16 April 2018).

[bibr4-1369148118772247] Agensky JC (2017) Recognizing religion: Politics, history, and the ‘long 19th century’. European Journal of International Relations 23: 729–755.

[bibr5-1369148118772247] Agnew J (2010) Deus vult: The geopolitics of the Catholic Church. Geopolitics 15: 39–61.

[bibr6-1369148118772247] Allen JJR (2013) Papabile of the day: The men who could be Pope. National Catholic Reporter, 10 3 Available at: https://goo.gl/Ar1Bwi (accessed 16 April 2018).

[bibr7-1369148118772247] Allen JJR (2014) Korea trip full of promise and peril for a ‘Peace Pope’. The Boston Globe, 9 8 Available at: http://goo.gl/eGLxIG (accessed 16 April 2018).

[bibr8-1369148118772247] Allen JL (2014) The Catholic Church: What Everyone Needs to Know. Oxford; New York: Oxford University Press.

[bibr9-1369148118772247] Alvarez D (1989) The professionalization of the papal diplomatic service, 1909–1967. The Catholic Historical Review 75(2): 233–248.

[bibr10-1369148118772247] Araujo RJ Lucal JA (2004a) A forerunner for international organizations: The Holy See and the community of Christendom: With special emphasis on the medieval papacy. Journal of Law and Religion 20(2): 305–350.

[bibr11-1369148118772247] Araujo RJ Lucal JA (2004b) Papal Diplomacy and the Quest for Peace. Ann Arbor, MI: Sapientia Press of Ave Maria University.

[bibr12-1369148118772247] Araujo RJ Lucal JA (2010) Papal Diplomacy and the Quest for Peace: The United Nations from Pius XII to Paul V. Philadelphia, PA: Saint Joseph’s University Press.

[bibr13-1369148118772247] Barbato M (2016) Legionen des Papstes. Zeitschrift Für Politikwissenschaft 26(4): 375–396.

[bibr14-1369148118772247] Barbato MP (2013) A state, a diplomat, and a transnational Church: The multi-layered actorness of the Holy See. Perspectives 21(2): 27–48.

[bibr15-1369148118772247] Barker JC (2006) The Protection of Diplomatic Personnel. Aldershot; Burlington, VT: Ashgate.

[bibr16-1369148118772247] Bátora J Hynek N (2014) Fringe Players and the Diplomatic Order: The New Heteronomy? Basingstoke; New York: Palgrave Macmillan.

[bibr17-1369148118772247] Bessner D Guilhot N (2015) How realism Waltzed off: Liberalism and decisionmaking in Kenneth Waltz’s Neorealism. International Security 40(2): 87–118.

[bibr18-1369148118772247] Bull H (2002) The Anarchical Society: A Study of Order in World Politics. New York: Columbia University Press.

[bibr19-1369148118772247] Bull H Watson A (1984) The Expansion of International Society. Oxford: Oxford University Press.

[bibr20-1369148118772247] Byrnes TA (2001) Transnational Catholicism in Postcommunist Europe. Lanham, MD: Rowman & Littlefield.

[bibr21-1369148118772247] Byrnes TA (2017) Sovereignty, supranationalism, and soft power: The Holy See in international relations. The Review of Faith & International Affairs 15(4): 6–20.

[bibr22-1369148118772247] Cahill L (2017) The realism of Holy See foreign policy. Available at: http://www.e-ir.info/2017/02/27/the-realism-of-holy-see-foreign-policy/ (accessed 16 April 2018).

[bibr23-1369148118772247] Cardinale HE (1976) The Holy See and the International Order. Toronto, ON, Canada: Macmillan of Canada.

[bibr24-1369148118772247] Casaroli AK (1981) Der Heilige Stuhl und die Völkergemeinschaft. Berlin: Duncker & Humblot.

[bibr25-1369148118772247] Chong A Troy J (2011) A universal sacred mission and the universal secular organisation: The Holy See and the United Nations. Politics, Religion, & Ideology 12(3): 335–354.

[bibr26-1369148118772247] Cismas I (2014) Religious Actors and International Law. Oxford: Oxford University Press.

[bibr27-1369148118772247] Constantinou CM (2013) Between statecraft and humanism: Diplomacy and its forms of knowledge. International Studies Review 15(2): 141–162.

[bibr28-1369148118772247] Constantinou CM McConnell F Cornago N (2016) Transprofessional diplomacy. Brill Research Perspectives in Diplomacy and Foreign Policy 1(4): 1–66.

[bibr29-1369148118772247] Conway JS (1979) Vatican diplomacy today: The legacy of Paul VI. International Journal 34(3): 457–474.

[bibr30-1369148118772247] Coriden JA Green TJ Heintschel DE (1985) The Code of Canon Law: A Text and Commentary. New York: Paulist Press.

[bibr31-1369148118772247] Cortright D (2008) Peace: A History of Movements and Ideas. Cambridge: Cambridge University Press.

[bibr32-1369148118772247] Der Derian J (1987a) Mediating estrangement: A theory for diplomacy. Review of International Studies 13(2): 91–110.

[bibr33-1369148118772247] Der Derian J (1987b) On Diplomacy: A Genealogy of Western Estrangement. Oxford; New York: Blackwell.

[bibr34-1369148118772247] Diez T (2017) Diplomacy, papacy, and the transformation of international society. The Review of Faith & International Affairs 15(4): 31–38.

[bibr35-1369148118772247] Essig AM Moore JL (2009) U.S.–Holy See diplomacy: The establishment of formal relations, 1984. Catholic History Review 95(4): 741–764.

[bibr36-1369148118772247] Feldkamp MF (2010) Geheim und Effektiv: Über 1000 Jahre Diplomatie der Päpste. Augsburg: Sankt Ulrich.

[bibr37-1369148118772247] Ferrara P (2015) The concept of periphery in Pope Francis’ discourse: A religious alternative to globalization? Religions 6(1): 42–57.

[bibr38-1369148118772247] Filoni F (2009) L’église dans la terre d’Abraham: Du diocèse de Babylone des Latins à la Nonciature apostolique en Iraq. Paris: Les Editions du Cerf.

[bibr39-1369148118772247] Fitzgerald T (2011) Religion and Politics in International Relations: The Modern Myth. New York: Continuum.

[bibr40-1369148118772247] Franco M (2013) The possible revolution of Pope Francis. Survival 55(6): 115–122.

[bibr41-1369148118772247] Franco M Flamini R (2008) Parallel Empires: The Vatican and the United States–Two Centuries of Alliance and Conflict. New York: Doubleday.

[bibr42-1369148118772247] Gaetan V (2015) The political Pope: How Francis was thrust into the world’s most intractable conflicts. Foreign Affairs, 25 9 Available at: https://goo.gl/efA2tc (last accessed 16 April 2018).

[bibr43-1369148118772247] Gaetan V (2017) Why Trump and Francis diverge on Saudi Arabia: The Vatican opposes Riyadh’s regional dominance. Foreign Affairs, 2 6 Available at: https://goo.gl/mHujIp (accessed 16 April 2018).

[bibr44-1369148118772247] Gallagher CR (2008) Vatican Secret Diplomacy: Joseph P. Hurley and Pope Pius XII. New Haven, CT: Yale University Press.

[bibr45-1369148118772247] Gould-Davies N (2013) The intimate dance of diplomacy: In praise of practice. International Affairs 89(6): 1459–1467.

[bibr46-1369148118772247] Graham RA (1959) Vatican Diplomacy: A Study of Church and State on the International Plane. Princeton, NJ: Princeton University Press.

[bibr47-1369148118772247] Grant TD (1999) The Recognition of States: Law and Practice in Debate and Evolution. Westport, CT: Praeger.

[bibr48-1369148118772247] Green DM (2014) Introduction to the English School in international studies. In: Navari C Green DM (eds) Guide to the English School in International Studies. Hoboken, NJ: Wiley-Blackwell, pp.1–6.

[bibr49-1369148118772247] Hall RB (1997) Moral authority as a power resource. International Organization 51(4): 591–622.

[bibr50-1369148118772247] Hanson EO (1987) The Catholic Church in World Politics. Princeton, NJ: Princeton University Press.

[bibr51-1369148118772247] Ivereigh A (2015) The Great Reformer: Francis and the Making of a Radical Pope. New York: Atlantic Books.

[bibr52-1369148118772247] Johnston D (2003) Faith-Based Diplomacy: Trumping Realpolitik. Oxford: Oxford University Press.

[bibr53-1369148118772247] Jones G Mackenzie J (2015) Pope Francis Extends Agenda of Change to Vatican Diplomacy. Toronto, ON, Canada: Thomson Reuters.

[bibr54-1369148118772247] Jönsson C Hall M (2005) Essence of Diplomacy. Basingstoke; New York: Palgrave Macmillan.

[bibr55-1369148118772247] Kempe F (1990) Divorcing the Dictator: America’s Bungled Affair with Noriega. London: I.B. Tauris.

[bibr56-1369148118772247] Kent PC Pollard JF (1994a) A diplomacy unlike any other: Papal diplomacy in the nineteenth and twentieth centuries. In: Kent PC Pollard JF (eds) Papal Diplomacy in the Modern Age. Westport, CT: Praeger, pp.11–21.

[bibr57-1369148118772247] Kent PC Pollard JF (1994b) Papal Diplomacy in the Modern Age. Westport, CT: Praeger.

[bibr58-1369148118772247] Kerr P Wiseman G (2013) Diplomacy in a Globalizing World: Theories and Practices. New York: Oxford University Press.

[bibr59-1369148118772247] Kissinger H (1995) Diplomacy. New York: Simon & Schuster.

[bibr60-1369148118772247] Kissinger H (2014) World Order: Reflections on the Character of Nations and the Course of History. London: Penguin Books.

[bibr61-1369148118772247] Köck HF (1975) Die Völkerrechtliche Stellung des Heiligen Stuhls: Dargestellt an Seinen Beziehungen zu Staaten und Internationalen Organisationen. Berlin: Duncker & Humblot.

[bibr62-1369148118772247] Koller A (1998) Kurie und Politik: Stand und Perspektiven der Nuntiaturberichtsforschung. Tübingen: Niemeyer.

[bibr63-1369148118772247] Kracht H-J (2011) Diplomatenausbildung des Heiligen Stuhles: 300 Jahre – von der ‘Accademia degli ecclesiastici nobili’ zur “Pontificia Accademia ecclesiastica.” Versuch eines historischen Einstiegs. In: Finger H Haas R Scheidgen H-J Trippen N (eds) Ortskirche und Weltkirche in der Geschichte: Kölnische Kirchengeschichte zwischen Mittelalter und Zweitem Vatikanum Festgabe für Norbert Trippen zum 75. Geburtstag. Köln: Böhlau Verlag, pp.969–995.

[bibr64-1369148118772247] Kurth J (1993) The Vatican’s foreign policy. The National Interest 32: 30–52.

[bibr65-1369148118772247] Laudy M (2000) The Vatican mediation of the Beagle Channel dispute: Crisis intervention and forum building. In: Greenberg MC Barton JH McGuinness ME (eds) Words Over War: Mediation and Arbitration to Prevent Deadly Conflict. Lanham, MD: Rowman & Littlefield, pp.293–320.

[bibr66-1369148118772247] Leustean LN (2013) Roman Catholicism, diplomacy, and the European communities, 1958–1964. Journal of Cold War Studies 15(1): 53–77.

[bibr67-1369148118772247] Little R (2011) Britain’s response to the Spanish Civil War: Investigating the implications of foregrounding practice for English School thinking. In: Adler E Pouliot V (eds) International Practices. Cambridge; New York: Cambridge University Press, pp.174–199.

[bibr68-1369148118772247] McLarren K Stahl B (2015) ‘Hybrid actors’ – religion and the shift towards a world society. In: Proceedings of the ECPR general conference, University of Montreal, Montreal, QC, Canada, 26–29 August.

[bibr69-1369148118772247] Macomber WB (1975) The Angels’ Game: A Handbook of Modern Diplomacy. New York: Stein and Day.

[bibr70-1369148118772247] Marshall K (2013) Global Institutions of Religion: Ancient Movers, Modern Shakers. London; New York: Routledge.

[bibr71-1369148118772247] Martens K (2006) The position of the Holy See and the Vatican City state in international relations. University of Detroit Mercy Law Review 83(5): 729–760.

[bibr72-1369148118772247] Mastrolilli P (2017) ‘The Vatican is like us Americans. A global power able to influence the world’: Dispatches sent to Washington before Bergoglio settled in. The first evaluations on the Pope: He is a conservative; he will not change the doctrine of the Church. La Stampa, 27 6 Available at: https://goo.gl/B8aC3A (accessed 16 April 2018).

[bibr73-1369148118772247] May S Wilson EK Baumgart-Ochse C et al (2014) The religious as political and the political as religious: Globalisation, post-secularism and the shifting boundaries of the sacred. Politics, Religion & Ideology 15(3): 331–346.

[bibr74-1369148118772247] Mayall J (2007) Introduction. In: Sharp P Wiseman G (eds) The Diplomatic Corps as an Institution of International Society. Basingstoke: Palgrave Macmillan, pp.1–12.

[bibr75-1369148118772247] Melnyk RA (2009) Vatican Diplomacy at the United Nations: A History of Catholic Global Engagement. Lewiston, NY: Edwin Mellen Press.

[bibr76-1369148118772247] Meyer JW Boli J Thomas GM et al (1997) World society and the nation-state. American Journal of Sociology 103(1): 144–181.

[bibr77-1369148118772247] Mitzen J (2015) From representation to governing: Diplomacy and the constitution of international public power. In: Sending OJ Pouliot V Neumann IB (eds) Diplomacy and the Making of World Politics. Cambridge: Cambridge University Press, pp.111–139.

[bibr78-1369148118772247] Morgenthau HJ (1956) Politics among Nations: The Struggle for Power and Peace, 2nd edn. New York: Alfred A. Knopf.

[bibr79-1369148118772247] Morgenthau HJ (1978) Politics among Nations: The Struggle for Power and Peace, 5th edn. New York: Alfred A. Knopf.

[bibr80-1369148118772247] Morss JR (2016) The international legal status of the Vatican/Holy See complex. European Journal of International Law 26(4): 927–946.

[bibr81-1369148118772247] Murphy FX (1974) Vatican politics: Structure and function. World Politics 26(4): 542–559.

[bibr82-1369148118772247] Navari C (2009) Introduction: Methods and methodology in the English School. In: Navari C (ed.) Theorising International Society: English School Methods. Basingstoke: Palgrave Macmillan, pp.1–20.

[bibr83-1369148118772247] Navari C (2011) The concept of practice in the English School. European Journal of International Relations 17(4): 611–630.

[bibr84-1369148118772247] Neale PR (1998) The bodies of Christ as international bodies: The Holy See, wom(b)an and the Cairo Conference. Review of International Studies 24(1): 101–118.

[bibr85-1369148118772247] Neumann IB (2002) Returning practice to the linguistic turn: The case of diplomacy. Millennium: Journal of International Studies 31(3): 627–651.

[bibr86-1369148118772247] Neumann IB (2003) The English School on diplomacy: Scholarly promise unfulfilled. International Relations 17(3): 341–369.

[bibr87-1369148118772247] Neumann IB (2010) Diplomacy and diplomats. In: Denemark RA (ed.) The International Studies Encyclopedia. Malden, MA: Wiley-Blackwell.

[bibr88-1369148118772247] Neumann IB (2011) ‘Religion in sort of a global sense’: The relevance of religious practices for political community in Battlestar Galactica and beyond. Journal of Contemporary Religion 26(3): 387–401.

[bibr89-1369148118772247] Neumann IB (2012) Euro-centric diplomacy: Challenging but manageable. European Journal of International Relations 18(2): 299–321.

[bibr90-1369148118772247] Nicolson H (1977) The Evolution of Diplomatic Method, Being the Chichele Lectures Delivered at the University of Oxford in November 1953. Westport, CT: Greenwood Press.

[bibr91-1369148118772247] Oliveri M (1982) Natura e funzioni dei legati pontifici nella storia e nel contesto ecclesiologico del Vaticano II. Rome: Libreria Editrice Vaticana.

[bibr92-1369148118772247] Parmelee J (1989) Nuncio urging Noriega to leave, Vatican says. The Washington Post, 30 12Available at: https://goo.gl/KgeqXt (accessed 16 April 2018).

[bibr93-1369148118772247] Parolin P (2017) Preface: The Holy See’s diplomatic mission in today’s world. In: Tomasi SM (ed.) The Vatican in the Family of Nations: Diplomatic Actions of the Holy See at the UN and Other International Organizations in Geneva. Cambridge; New York: Cambridge University Press, pp.ix–xvi.

[bibr94-1369148118772247] Petito F Thomas SM (2015) Encounter, dialogue, and knowledge: Italy as a special case of religious engagement in foreign policy. The Review of Faith & International Affairs 13(2): 40–51.

[bibr95-1369148118772247] Plöger K (2005) England and the Avignon Popes: The Practice of Diplomacy in Late Medieval Europe. London: Legenda.

[bibr96-1369148118772247] Pontificia Accademia Ecclesiastica (2003) Terzo Centenario (1701–2001). Rome: Libreria Editrice Vaticana.

[bibr97-1369148118772247] Pope Francis (2013) Address of Pope Francis to the community of the Pontifical Ecclesiastical Academy. Available at: https://goo.gl/Yf9QJf (accessed 16 April 2018).

[bibr98-1369148118772247] Pope John XXIII (1966) Mission to France: Memoirs of a Nuncio 1944–1953. New York: McGraw-Hill Book Company.

[bibr99-1369148118772247] Pouliot V Cornut J (2015) Practice theory and the study of diplomacy: A research agenda. Cooperation and Conflict 50(3): 297–315.

[bibr100-1369148118772247] Povoledo E (2013) Pope appeals for more interreligious dialogue. The New York Times, 22 3 Available at: http://goo.gl/P6U2Ag (accessed 16 April 2018).

[bibr101-1369148118772247] Princen T (1987) International mediation–the view from the Vatican: Lessons from mediating the Beagle Channel dispute. Negotiation Journal 3(4): 347–366.

[bibr102-1369148118772247] Princen T (1992) Mediation by a transnational organization: The case of the Vatican. In: Bercovitch J (ed.) Mediation in International Relations: Multiple Approaches to Conflict Management. Basingstoke: Macmillan, pp.149–175.

[bibr103-1369148118772247] Prudhomme C (2004) L’Académie pontificale ecclésiastique et le service du Saint-Siège. Mélanges de l’école française de Rome 116(1): 61–89.

[bibr104-1369148118772247] Radio Vatikan (2017) Papst besucht zukünftige Diplomaten. Available at: https://goo.gl/kYWqHo (accessed 28 May 2017).

[bibr105-1369148118772247] Rennie KR (2013) The Foundations of Medieval Papal Legation. Basingstoke: Palgrave Macmillan.

[bibr106-1369148118772247] Riccards MP (1998) Vicars of Christ: Popes, Power, and Politics in the Modern World. New York: Crossroad.

[bibr107-1369148118772247] Roberts I Satow EM (2011) Satow’s Diplomatic Practice. Oxford; New York: Oxford University Press.

[bibr108-1369148118772247] Rooney F (2013) The Global Vatican: An inside Look at the Catholic Church, World Politics, and the Extraordinary Relationship between the United States and the Holy See. Lanham, MD: Rowman & Littlefield.

[bibr109-1369148118772247] Rosenthal A (1990) Noriega’s surrender: Chronology; Vatican issues an ultimatum and a general takes a walk. The New York Times, 5 1 Available at: https://goo.gl/TCtBpV (accessed 16 April 2018).

[bibr110-1369148118772247] Rother L (1990) The Noriega case: Panama city; Papal envoy asserts psychology, not ultimatum, Swayed Noriega. The New York Times, 6 1 Available at: https://goo.gl/ikfMtb (accessed 16 April 2018).

[bibr111-1369148118772247] Rotte R (2007) Die Außen- und Friedenspolitik des Heiligen Stuhls: Eine Einführung.Wiesbaden: VS Verlag für Sozialwissenschaften (GWV).

[bibr112-1369148118772247] Ryall D (1998) How many divisions? The modern development of Catholic international relations. International Relations 14(2): 21–34.

[bibr113-1369148118772247] Ryall D (2001) The Catholic Church as a transnational actor. In: Josselin D Wallace W (eds) Non-state Actors in World Politics. Basingstoke: Palgrave Macmillan, pp.41–58.

[bibr114-1369148118772247] Schelkens K (2011) Vatican diplomacy after the Cuban Missile Crisis: New light on the release of Josyf Slipyj. The Catholic Historical Review 97(4): 679–712.

[bibr115-1369148118772247] Sending OJ (2011) United by difference: Diplomacy as a thin culture. International Journal 66(3): 643–659.

[bibr116-1369148118772247] Sending OJ Pouliot V Neumann IB (2015) Introduction. In: Sending OJ Pouliot V Neumann IB (eds) Diplomacy and the Making of World Politics. Cambridge: Cambridge University Press, pp.1–28.

[bibr117-1369148118772247] Sharp P (1997) Who needs diplomats? The problem of diplomatic representation. International Journal 52: 609–634.

[bibr118-1369148118772247] Sharp P (2009) Diplomatic Theory of International Relations. Cambridge: Cambridge University Press.

[bibr119-1369148118772247] Sharp P Wiseman G (2007) The Diplomatic Corps as an Institution of International Society. Basingstoke: Palgrave Macmillan.

[bibr120-1369148118772247] Sheikh MK (2012) How does religion matter? Pathways to religion in international relations. Review of International Studies 38(02): 365–392.

[bibr121-1369148118772247] Sodano A (2000) The Holy See’s presence in international affairs. Seton Hall Journal of Diplomacy and International Relations 2: 87–91.

[bibr122-1369148118772247] Sofer S (1997) The diplomat as a stranger. Diplomacy & Statecraft 8(3): 179–186.

[bibr123-1369148118772247] Sommeregger A (2011) Soft Power und Religion: Der Heilige Stuhl in den Internationalen Beziehungen.Wiesbaden: VS Verlag für Sozialwissenschaften.

[bibr124-1369148118772247] The Economist (2007) Papal diplomacy: God’s ambassadors. The Economist, 19 7 Available at: http://www.economist.com/node/9516461 (accessed 16 April 2018).

[bibr125-1369148118772247] Thomas SM (2000) Taking religious and cultural pluralism seriously: The global resurgence of religion and the transformation of international society. Millennium 29(3): 815–841.

[bibr126-1369148118772247] Thomas SM (2010) A globalized God: Religion’s growing influence in international politics. Foreign Affairs 89(6): 93–101.

[bibr127-1369148118772247] Tomasi SM (2017) The Vatican in the Family of Nations: Diplomatic Actions of the Holy See at the UN and Other International Organizations in Geneva. Cambridge; New York: Cambridge University Press.

[bibr128-1369148118772247] Troy J (2008) Faith-based diplomacy under examination. Hague Journal of Diplomacy 3(3): 209–231.

[bibr129-1369148118772247] Troy J (2010) Die soft power des Heiligen Stuhls: Legionen auf unsichtbarer Parade zwischen internationaler Gesellschaft und Weltgesellschaft. Zeitschrift Für Außen-und Sicherheitspolitik 3(4): 489–511.

[bibr130-1369148118772247] Turina I (2015) Centralized globalization: The Holy See and human mobility since World War II. Critical Research on Religion 3(2): 189–205.

[bibr131-1369148118772247] Vallier I (1971) The Roman Catholic Church: A transnational actor. International Organization 25(3): 479–502.

[bibr132-1369148118772247] Watson A (1991) Diplomacy: The Dialogue between States. London; New York: Routledge.

[bibr133-1369148118772247] Wee S-L (2014) Special Report: The Bishop Who Stood Up to China. Available at: http://goo.gl/K5IHW5 (accessed 16 April 2018).

[bibr134-1369148118772247] Wight M (1966) Why is there no international theory? In: Butterfield H Wight M (eds) Diplomatic Investigations: Essays in the Theory of International Politics. London: Allen & Unwin, pp.17–34.

[bibr135-1369148118772247] Wight M (1979) Power Politics. London: Pelincan Books.

[bibr136-1369148118772247] Wight M (1991) International Theory: The Three Traditions. Leicester: Leicester University Press.

[bibr137-1369148118772247] Wight M (1997) System of States. Leicester: Leicester University Press.

[bibr138-1369148118772247] Wilson EK (2010) Beyond dualism: Expanded understandings of religion and global justice. International Studies Quarterly 54(3): 733–754.

[bibr139-1369148118772247] Wilson EK (2014) Theorizing religion as politics in postsecular international relations. Politics, Religion & Ideology 15(3): 347–365.

[bibr140-1369148118772247] Wolfers A (1962) Discord and Collaboration: Essays on International Politics. Baltimore, MD: Johns Hopkins University Press.

[bibr141-1369148118772247] Wuthe P (2002) Für Menschenrechte und Religionsfreiheit in Europa: Die Politik des Heiligen Stuhls in der KSZE/OSZE. Stuttgart: Kohlhammer.

[bibr142-1369148118772247] Yardley J (2014) Under Francis, a bolder vision of Vatican diplomacy emerges. The New York Times, 18 12 Available at: https://goo.gl/Zt4CJm (accessed 16 April 2018).

